# Magnesium: A Defense Line to Mitigate Inflammation and Oxidative Stress in Adipose Tissue

**DOI:** 10.3390/antiox13080893

**Published:** 2024-07-24

**Authors:** Roberta Cazzola, Matteo Della Porta, Gabriele Piuri, Jeanette A. Maier

**Affiliations:** Department of Biomedical and Clinical Sciences, University of Milano, 20174 Milan, Italy; roberta.cazzola@unimi.it (R.C.); matteo.dellaporta@guest.unimi.it (M.D.P.); gabriele.piuri@me.com (G.P.)

**Keywords:** hypomagnesemia, antioxidants, calcium antagonism, low-grade chronic inflammation, obesity

## Abstract

Magnesium (Mg) is involved in essential cellular and physiological processes. Globally, inadequate consumption of Mg is widespread among populations, especially those who consume processed foods, and its homeostasis is impaired in obese individuals and type 2 diabetes patients. Since Mg deficiency triggers oxidative stress and chronic inflammation, common features of several frequent chronic non-communicable diseases, interest in this mineral is growing in clinical medicine as well as in biomedicine. To date, very little is known about the role of Mg deficiency in adipose tissue. In obesity, the increase in fat tissue leads to changes in the release of cytokines, causing low-grade inflammation and macrophage infiltration. Hypomagnesemia in obesity can potentiate the excessive production of reactive oxygen species, mitochondrial dysfunction, and decreased ATP production. Importantly, Mg plays a role in regulating intracellular calcium concentration and is involved in carbohydrate metabolism and insulin receptor activity. This narrative review aims to consolidate existing knowledge, identify research gaps, and raise awareness of the critical role of Mg in supporting adipose tissue metabolism and preventing oxidative stress.

## 1. Introduction

Adipose tissue (AT) has historically been classified into two types: white adipose tissue (WAT) and brown adipose tissue (BAT). In addition, recently, beige adipose tissue has been described [1]. WAT represents most AT in adult humans and is the body’s main energy storage. At the same time, BAT dissipates energy as a defense against cold and maintains energy balance for the whole body. Beige adipose tissue is located within WAT but shares similar features with BAT. WAT comprises many cell types, such as adipocytes (the most abundant), preadipocytes, fibroblasts, stem cells, macrophages, and capillary endothelial cells (ECs). Of note, an intense reciprocal dynamic communication exists between ECs and adipocytes. Endothelial transfer of plasma constituents and biological signals via secretory signaling molecules and microvesicles to the adipocytes [2] is crucial for metabolic homeostasis. Conversely, adipocytes release various bioactive molecules that influence endothelial function. The role of adipose tissues in modulating vascular homeostasis is attracting more and more attention, because most blood vessels are surrounded by a functionally specialized aggregate of AT, termed perivascular adipose tissue (PVAT). PVAT is an established regulator of vascular function through its release of gases, such as nitric oxide (NO) and hydrogen sulfide, and adipokines. While in physiological conditions, PVAT is vasculo-protective, its dysregulation contributes to vascular dysfunction since it releases inflammatory mediators that readily promote oxidative stress (OS) and the acquisition of an inflammatory phenotype of vascular cells [3].

Magnesium (Mg) deficiency is one of the many factors involved in promoting OS and inflammation. Globally, the intake of Mg is inadequate, and this trend is widespread across populations. Subclinical Mg deficiency has been observed mainly in populations that consume processed foods, and it has also been observed in obese individuals [4]. In obesity, the expansion of AT results in the alteration of its secretion of cytokines, initiating a cascade of metabolic changes that favor macrophage infiltration mediated and sustained by low-grade chronic inflammation (LGCI). Moreover, the pro-inflammatory phenotype of hypertrophic/hyperplastic adipocytes in obesity promotes EC activation, contributing to LGCI [2]. Proper Mg intake can directly limit OS by reducing pro-oxidants and enhancing antioxidant capacity. It can also indirectly reduce inflammation [5,6].

We performed a literature review using MEDLINE, PubMed, EMBASE, and Web of Science to assess the impact of Mg on OS and inflammation of AT, focusing on obese subjects. We meticulously analyzed the full-text articles, selecting the most pertinent studies for inclusion in this review.

## 2. Oxidative Stress, Inflammation, and AT Dysfunction

Our understanding of the role of reactive oxygen species (ROS) in cellular biology has evolved considerably, revealing their role as essential regulators in the cellular signaling network. In physiological conditions, homeostatic ROS are secondary messengers in various intracellular signaling pathways, involving programmed cell death or necrosis, gene expression regulation, and innate and adaptive immune responses [7]. In addition, ROS are implicated in body weight control because they are crucial for the response of hypothalamic neurons to fluctuations in levels of bodily metabolic fuels such as glucose, free fatty acids, and amino acids [8].

ROS are generated during several biochemical processes, such as mitochondrial ATP synthesis, the activity of NADPH oxidases, nitric oxide synthases (NOSs) and microsomal cytochrome P450 oxidases, Fenton reaction, peroxisomal β-oxidation, prostaglandin synthesis, and others [9]. However, mitochondria are the main source of cellular ROS [10]. The rate of ROS production by isolated mitochondria is dependent on the metabolic state and is inversely related to the coupling between the respiratory chain and ATP synthesis [11,12]. Adipocytes adapt to dynamic changes in ROS levels and use them as second messengers. As an example, hydrogen peroxide has been found to mimic the action of insulin in adipocytes [9].

ROS accumulation directly contributes to the pathophysiology of several chronic inflammatory diseases, causing lipid peroxidation, DNA damage, protein oxidation, irreversible mitochondrial damage, inadequate ATP production, and indirectly, activating the nuclear factor-κB (NF-κB) pathway and, therefore, inflammation [13]. Inflammation and oxidative stress are two related processes: one can promote the other, leading to a toxic feedback system. A rich antioxidant arsenal tightly regulates ROS production. The body’s antioxidant defenses include a variety of water-soluble and fat-soluble compounds, such as enzymes, proteins, glutathione, urate, vitamins C and E, and beta-carotene, which work together to neutralize the effects of pro-oxidants. Antioxidant enzymes include catalase (CAT), glutathione reductase (GR), thioredoxin reductase (TrxR), heme oxygenase-1 (HO-1), superoxide dismutase (SOD), glutathione peroxidase (GPx), peroxiredoxin (Prx), paraoxonases (PON), and NAD(P)H: quinone oxidoreductase 1 (NQO1). However, the prolonged and uncontrolled production of ROS can overcome the body’s antioxidant defense system, generating OS. In obesity, systemic OS is due to excessive superoxide generation from nicotinamide adenine dinucleotide phosphate (NADPH) oxidase (NOX), uncoupled endothelial nitric oxide synthase (eNOS), and reduced antioxidant defense [4]. Exposure to OS can lead to the damage of proteins by promoting their carbonylation. This process is an irreversible non-enzymatic modification that can cause protein dysfunction and aggregation. In the case of obesity, higher levels of carbonylated proteins appear to be linked to mitochondrial dysfunction in adipocytes, which may be relevant to the development of insulin resistance (IR) and type 2 diabetes [9].

In obesity, AT expands by a combination of an increase in adipocyte size and number. There may be a difference in the rates of growth between AT and vascularity, leading to reduced blood flow in the area. This can create an oxygen gradient and cause a relatively low-oxygen environment in the growing AT [14]. Research has shown that this hypoxia can alter the expression and release of adipokines in human adipocytes because of an increased expression of the hypoxia-inducible factor 1 alpha [15] and intracellular calcium (Ca) concentration oscillations [14]. Increased intracellular Ca concentration regulates the hypoxia-induced release of leptin, vascular endothelial growth factor, and interleukin (IL)-6, while the release of adiponectin is NF-κB-dependent.

**Figure 1:**
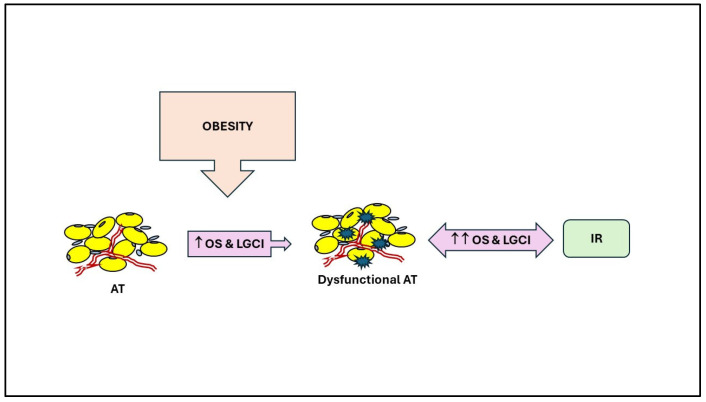
Relationships between AT dysfunction, oxidative stress, inflammation, and insulin resistance in obesity. LGCI: low-grade chronic inflammation; OS: oxidative stress; IR: insulin resistance; ↑: increase.

These adipokines are pro-oxidant and pro-inflammatory, except adiponectin. In normal-weight individuals, small adipocytes effectively store fatty acids as triglycerides (TG). Excessive caloric intake can lead to an overload of the metabolic system, causing an increase in TG storage and the enlargement of adipocytes. In addition, OS in the WAT disturbs its redox balance and impacts its function by impairing adipogenesis, inducing IR, and causing adipocyte hypertrophy [9] (Figure 1).

Hypertrophic adipocytes exhibit a decreased density of insulin receptors and an elevated level of β-3 adrenergic receptors. This facilitates the migration of monocytes to the visceral adipose stroma, initiating a proinflammatory cycle between adipocytes and monocytes, causing tissue dysfunction and contributing to obesity-related inflammation and comorbidities [16]. Physiologically, monocytes reach the adipose tissue during development, become resident AT macrophages, and regulate the metabolic activity of adipocytes and their precursors to maintain AT homeostasis and efficient function. Importantly, they display an anti-inflammatory M2 phenotype [17]. On the contrary, in obese individuals, AT macrophages tend to polarize to a pro-inflammatory M1 phenotype. M1 macrophages release chemokines, such as monocyte chemoattractant protein-1 (MCP-1), and pro-inflammatory cytokines, such as IL-1β, IL-6, and tumor necrosis factor-α (TNF-α), contributing to LGCI [18]. These cytokines not only promote their local proliferation but also recruit more macrophages and retain them in the AT to the point that the proportion of macrophages significantly increases in the adipose tissue of obese people [19]. Obesity-related metabolic inflammation in AT gradually impacts other organs through lipid and inflammatory mediators. The surplus of circulating TG and free fatty acids causes an accumulation of activated lipids in the muscle, disrupting functions such as mitochondrial oxidative phosphorylation and insulin-stimulated glucose transport, ultimately leading to peripheral IR and further potentiating OS [18]. In addition, nutrient overload alters AT metabolism towards increased lipid accumulation and glycolytic ATP synthesis in conjunction with decreased mitochondrial biogenesis. Overeating also activates inflammatory responses in the liver, skeletal muscle, pancreas, and hypothalamus, thus contributing to diminished insulin sensitivity and systemic IR in obese individuals [4]. In obesity, IR is often associated with hyperinsulinemia, increased visceral adiposity, metabolic dyslipidemia with high TG levels, low HDL cholesterol levels, and hypertension, features collectively referred to as metabolic syndrome (MetS). The cluster of conditions defining MetS increases the risk of coronary heart disease and type 2 diabetes [4].

## 3. Mg

Mg is a mineral macro-element that functions as a crucial signaling element and metabolite in cell physiology [20,21]. Mg participates in metabolizing lipids, proteins, carbohydrates, and nucleic acids [22]. Mg also mitigates the effects of OS and maintains cell membrane stability [23]. At the organ level, Mg is essential to maintain proper bone density and glucose tolerance, especially in diabetic patients, to relieve neurological and psychiatric disorders, to regulate blood pressure, to prevent ischemic heart disease and strokes, and to maintain an adequate skeletal muscular mass, principally in the elderly [22,24]. Mg should be consumed following the appropriate dietary recommendations to maintain these physiological functions. The recommended dietary allowance (RDA) for Mg in the USA is 310–320 and 410–420 mg per day for females and males, respectively, while in Europe, the European Food Safety Authority (EFSA) recommends 300 and 350 mg per day for women and men, respectively [24]. Globally, the reduction in dietary Mg intake is mainly due to (i) the effect of global warming that reduces Mg amounts in crops; (ii) the impact of cereals’ milling that drastically reduces Mg content in the grains (e.g., whole wheat flour 120 mg/100 g to refined wheat flour 20 mg/100 g of product); and (iii) the change in the dietary pattern of the global population from diets rich in vegetables, whole grains, fruit, and nuts rich in Mg to a Western dietary pattern rich in refined grains, sugars, saturated fats, additives and poor in veggies and fruits [24]. Mg dietary intake decreases with the increasing westernization of the dietary pattern worldwide. Beyond dietary intake, pre-existing pathological conditions (such as impaired gastrointestinal absorption, inflammatory bowel disease, colon cancer, and gastric bypass), diabetes mellitus, renal disorders and hydro-electrolyte imbalances, genetic factors, alcohol abuse, and the use of certain medications result in chronic Mg deficiency [25,26,27,28,29,30,31]. Aging is a significant risk factor for Mg deficiency. Despite stable Mg requirements across age groups [32,33], older adults do not consume enough Mg, regardless of gender and race [34]. Furthermore, aging reduces the efficiency of Mg absorption and active renal reabsorption [33]. In older adults, an imbalance in Mg can lead to greater susceptibility to age-related diseases like cardiovascular disease and diabetes but may also contribute to the aging process itself [35]. This is not surprising considering that Mg deficiency causes OS, which is one of the hallmarks of aging [36].

Also, strenuous physical activity can lead to Mg deficiency [37]. This factor should be considered when determining the appropriate Mg levels for active individuals. Exercise triggers a shift in Mg within the body tissues to support metabolic demands. Mg transits between plasma, adipocytes, and skeletal muscle during and post aerobic workouts. The extent of movement depends on cell energy level production. Following exercise, Mg moves back to the bloodstream from tissues by drawing from bone, muscle, and adipose tissue. Although tubular reabsorption mechanisms act to minimize urinary losses of Mg, post-workout urine Mg excretion rises. These changes affect Mg levels in various body fluids and tissues. Inadequate Mg intake, especially for athletes, can impair performance and worsen exercise effects [37]. As with all essential nutrients, Mg deficiency can be corrected by increasing Mg intake through diet and/or supplementation. To correct hypomagnesemia, supplementation of 250 to 600 mg per day of Mg, preferably in the form of organic Mg salts due to their higher bioavailability, is useful [18]. Even better, an increase in dietary Mg can help correct hypomagnesemia [24]. As an example, Mg enteric absorption from almonds is much higher compared to that from Mg supplements [38]. Accordingly, adolescents’ adherence to the Mediterranean diet, which is high in Mg, increases Mg levels and helps prevent obesity [39]. In addition, an Mg food source is more effective than supplements in decreasing all-cause mortality [40].

## 4. Mg Deficiency, Inflammation, and Oxidative Stress

**Figure 2:**
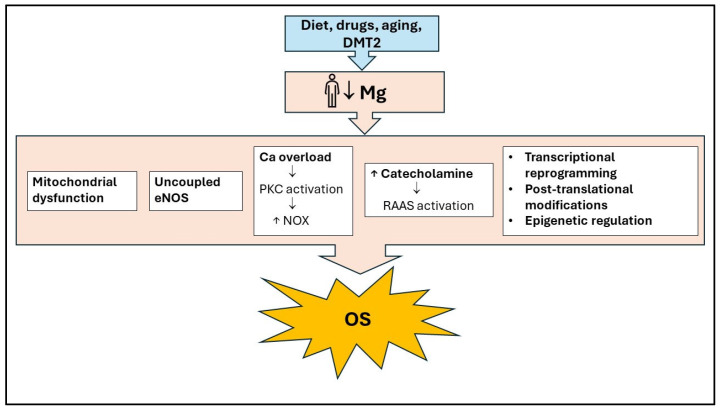
The main cellular mechanisms of Mg’s preventive action against oxidative stress. Mg deficiency leads to oxidative stress by causing mitochondrial dysfunction, abnormal calcium homeostasis, eNOS uncoupling, Renin-Angiotensin-Aldosterone System activation, and changes in transcription and translation processes. Ca: Calcium; DMT2: Diabetes Mellitus type 2; eNOS, Endothelial Nitric Oxide Synthase; OS: Oxidative Stress; NOX: NADPH oxidase; RAAS: Renin-Angiotensin-Aldosterone System; ↑: increase; ↓: decrease.

Mg deficiency causes excessive production of ROS, mainly due to mitochondrial dysfunction, abnormal calcium homeostasis, and activation of the renin-angiotensin-aldosterone system [6] (Figure 2). Furthermore, the antioxidant defense system is impaired, and ATP synthase is downregulated, causing a decrease in ATP production (Figure 1). In AT, Mg regulates intracellular Ca concentration by blocking the opening of the L-type Ca channel, which is controlled by the Mg-binding sites. The excess of intracellular Ca, in turn, results in the activation of Ca-dependent processes such as the release of inflammatory cytokines and activation of NOX by phosphorylation of protein kinase C (PKC), activation of NOS and calcium-dependent calmodulin complex, and hence increased OS [41] (Figure 2).

Intracellular Mg also acts as an essential cofactor of several enzymes involved in carbohydrate metabolism, regulating the activity of those that catalyze phosphorylation reactions and acting as part of the Mg–ATP complex, necessary for the action of enzymes that participate in glycolysis. Thus, the appropriate Mg concentration is essential for the tyrosine kinase activity of the insulin receptor and, therefore, for the autophosphorylation of the β subunit of this receptor and phosphorylation of its substrates [42].

The glutathione system constitutes the primary mechanism for mitigating OS within the body. Its activity is closely related to the redox state of other low-molecular-weight thiols, such as cysteine, cysteine-glycine, and homocysteine. This system operates at both intracellular and plasma levels, and the redox state of the plasma pool is in equilibrium with that of the intracellular pool. Glutathione is the primary intracellular antioxidant. It can be released from tissue, contributing to maintaining other thiols in the reduced form. The reducing power of water-soluble thiols is necessary to keep some fat-soluble antioxidants (such as vitamin E and coenzyme Q) in their active, reduced form. Mg is an obligatory cofactor in glutathione synthesis and all biosynthetic reactions involving ATP. Additionally, hypomagnesemia contributes to a reduction in the expression and activity of antioxidant enzymes (such as GPx, SOD, and CAT), leading to a decrease in cell and tissue concentrations of antioxidants and an increase in the production of the ROS hydrogen peroxide and superoxide anion by inflammatory cells [23].

The production of antioxidants, the expression of anti-oxidative enzymes, and AT metabolism have been reported to be regulated by circadian rhythms [43]. The circadian rhythm generally fluctuates in a daily cycle of about 24 h, a period of light and dark, in response to abiotic and biotic factors. The circadian clock is a network of molecular clocks in central and peripheral tissues that orchestrates biological processes in adapting to everyday environmental changes. The central circadian clock is in the hypothalamic suprachiasmatic nuclei, while the peripheral clocks are in other tissues, including the kidney, liver, blood vessels, and AT [43]. Accumulating data from both human and experimental animal models suggest that AT function and OS have an innate connection with the intrinsic biological clock [43]. Interestingly, daily Mg fluxes regulate cellular timekeeping and energy balance [44].

Recent studies have demonstrated that obesity leads to increased OS in both humans and animals [45]. The level of OS in the body was found to be directly associated with fat mass in both humans and rodents [46]. In obese individuals, markers of mitochondrial OS, such as protein carbonyls, lipid peroxidation products including malondialdehyde, and production of ROS, were elevated in AT [47]. Considering the various properties of Mg that contribute to maintaining metabolic and redox balance in AT, an adequate level of Mg in this tissue can prevent OS and its effects on obesity.

## 5. Role of Mg in Adipose Tissue: Preclinical Evidence

Mg deficiency in rodents contributes to accelerated catabolism, partly by generating insulin resistance, but the metabolic phenotype analysis has not been disclosed [48,49,50]. Controversies exist about the effect of an Mg-deficient diet on body mass. In some studies, Mg dietary restriction had no impact on body mass and adiposity index [49,50], while in others, a decrease in body mass was reported because of total lean body mass decrease without changes in total body fat. These findings were attributed to reduced appetite and decreased food consumption [51,52].

Table 1 summarizes the preclinical evidence on the role of Mg in AT.

An interesting study has highlighted how age affects AT in either Mg-deficient rats or those supplemented with Mg [53]. In rats fed a standard diet, the significant increase in AT weight observed during aging was related to a rise in both the size and number of adipocytes. In young rats, Mg deficiency did not change the size of the adipocytes but increased their number (30% more than the standard diet or supplementation), suggesting that a low Mg status contributes to improving the lipid storage capacity of AT. In aged rats, a Mg-deficient diet led to relative hypotrophy of adipocytes, counterbalanced by the rise in their number. In brief, inadequate Mg intake affected the size and number of fat cells in both young and aged rats.

Notably, Mg restriction abrogates weight gain in rats exposed to a sucrose-rich diet, mainly because de novo lipogenesis is reduced under Mg deficiency [54,55]. In this experimental model, low Mg intake exacerbates OS in sucrose-fed rats, leading to increased oxidative damage to unsaturated lipids in membranes and amino acids in proteins [55]. In parallel, the activities of the SOD, glutathione-S-transferase, and catalase decline considerably in animals under a low Mg/sucrose-rich diet [56]. Moreover, a low dietary Mg intake prevents high-fat diet-induced obesity in mice by enhancing the expression of genes involved in β oxidation and by elevating Ucp1 levels in BAT, with the consequent increased thermogenesis [57]. The two events are linked to elevated β3-adrenergic receptor expression in WAT and BAT.

Mg intake must be adequate from the early stages of development. Maternal Mg deficiency in mice irreversibly increases the visceral adiposity of the offspring, which shows higher expression of fatty acid synthase and fatty acyl transport protein 1, liver, and adipose tissue together with increased levels of plasma TNF-α [58].

**Figure 3:**
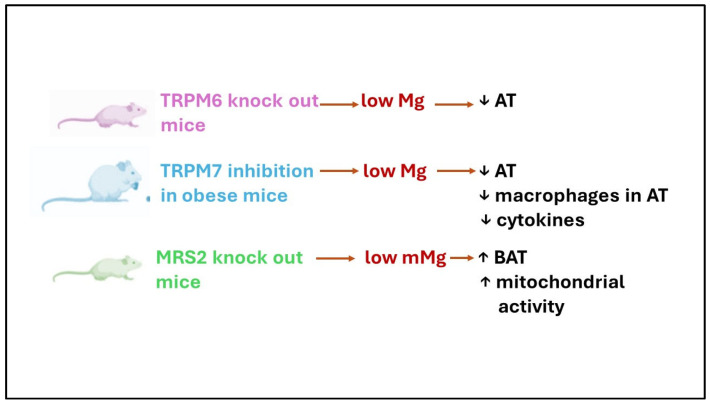
A summary of the effects of inhibiting certain Mg transporters/channels in mice. TRPM: transient receptor potential ion channels for melastatin; MRS2: mitochondrial RNA splicing 2; mMg: mitochondrial Mg; ↓: decrease; ↑: increase.

Interesting hints about the complex role of Mg in the AT derive from Mg transporter/channel knock-out mice (Figure 3). Transient receptor potential ion channel for melastatin (TRPM) 6, a kinase-coupled ion channel, is essential in the intestine for maintaining organismal Mg levels, while its closest relative, TRPM7, is considered indispensable for cellular Mg homeostasis. TRPM6^−/−^ mice, which develop severe hypomagnesemia because of deficient intestinal Mg uptake, are entirely devoid of abdominal and subcutaneous fat depots and present clear signs of catabolic metabolism [48]. In obese mice, pharmacological inhibition of TRPM7 reduces body weight by reducing the adipose mass and prevents insulin resistance (Figure 3). Of particular interest is the finding that the percentage of macrophage in WAT is significantly reduced, and this correlates with the downregulation of the pro-inflammatory cytokines IL1β and IL6 and the chemokine MCP-1 [59].

Similarly, serum levels of TNF-α, IL-6, IL-1β, and MCP-1 were upregulated in obese mice and reduced by the conditional knock-out of TRPM7 in the AT [60]. These results point to TRPM7 as the link between obesity and inflammation. Indeed, since low Mg upregulates TRPM7, many of the effects of Mg deficiency, including inflammation and OS, can be attributed to increased TRPM7 and the activation of its kinase domain [60]. A positive feedback loop exists between OS levels and TRPM7’s expression and function. Elevated OS increases TRPM7 expression [61,62], while TRPM7 overexpression induces the accumulation of intracellular ROS and inflammation [63,64]. In addition, since the promoter region of TRPM7 contains binding sites for NF-κB, LGCI upregulates TRPM7 [59].

Mitochondria are central to health and disease [65]. They are involved in cell metabolism and regulate ion homeostasis, cell growth, redox status, and cell signaling. Moreover, they are intracellular Mg stores [66]. Mg, pivotal for protein and ATP synthesis and various metabolic pathways, enters the mitochondria through the Mitochondrial RNA Splicing 2 (MRS2) channel anchored to the inner mitochondrial membrane [67]. MRS2^−/−^ mice on a Western diet do not gain weight and show enhanced mitochondrial activity and increased BAT [68]. Accordingly, the knockdown of MRS2 in human cells leads to decreased mitochondrial uptake of Mg and metabolic reprogramming [69].

**Table 1:** Preclinical evidence on the role of Mg in adipose tissue.

Study	Type of Animal	Treatment	Treatment Duration	Effects
Devaux et al. [53]	Male Sprague Dawley young rats (YR) vs. old rats (OR)	Diet with Mg deficiency	22 mo	YR adipocytes hyperplasia; OR hypotrophy of adipocytes
Boparai et al. [55]	Male Wistar rats	High sucrose (HS); low Mg (LM); HSLM	12 wk	HSLM ↑ of TBARS and PCO (plasma and liver)
Kurstjens et al. [57]	Male C57BL6/J mice	normal Mg Low-fat diet (NMLFD); NM High fat diet (NMHFD); LMLFD; LMHFD	17 wk	LM ameliorates HFD-induced obesity, fasting glucose ↓, insulin sensitivity ↑; absence of liver steatosis; ↑ BAT Ucp1 m-RNA expression, and higher body temperature
Zhong et al. [59]	Flox and ATKO mice	HFD; TRPM7 inhibition	16 wk	ATKO have less body weight than Flox, ↓ % of macrophage in WAT, IL-1β, IL-6, MCP-1
Madaris et al. [68]	Male WT and MRS2^−/−^ KO mice	HFD; Western Diet (WD)	12 mo	MRS2^−/−^ KO in WD no weight gain and ↑ mitochondrial activity and BAT
Choudary et al. [56]	Male Wistar rats	LM; HS; HSLM	3 mo	↓ SOD, catalase and GST in HSLM

ATKO = adipocyte-specific TRPM7 knock-out; BAT = brown adipose tissue; GST = glutathione-S-transferase; HFD = high fat diet; HS = high sucrose; IL = interleukin; KO = knock-out; LFD = low fat diet; LM = low Mg; MCP-1 = monocyte chemoattractive protein 1; MRS2 = mitochondrial RNA splicing 2; NM = normal Mg; OR = old rats; PCO = protein carbonyls; SOD = superoxide dismutase; TBARS = thiobarbituric acid reactive substances; TRPM7 = transient receptor potential ion channels for melastatin; WAT = white adipose tissue; WD = western diet; WT = wild-type; YR = young rats; ↑ = increase; ↓ = decrease; wk = weeks; mo = months.

Very little is known about the effect of low Mg on cultured adipocytes. Mg deficiency impairs insulin-dependent glucose metabolism in isolated rat adipocytes [70]. In mature 3T3-L1 adipocytes, Mg deficiency diminishes GLUT4 translocation and decreases glycolysis upon insulin stimulation because of the lack of Akt activation [71]. Currently, no data are available about redox balance and the secretion of cytokines and adipokines in Mg-deficient adipocytes. In an in vitro model of brown adipocyte differentiation, increasing extracellular Mg tends to enhance the expression of PR domain containing 16 (PRDM 16) and PPARγ coactivator 1-alpha (PGC1-α), two adipogenic factors, thus suggesting the inhibition of brown adipocyte differentiation through a calcium antagonistic effect [72].

**Figure 4:**
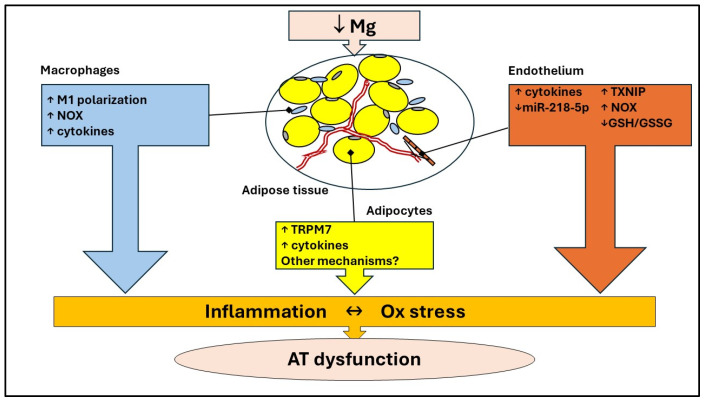
Crosstalk between different cell types in AT. In adipocytes, Mg deficiency upregulates TRMP7, which appears to elevate cytokine levels. Moreover, low Mg promotes a pro-oxidant and pro-inflammatory phenotype in endothelial cells and induces M1 macrophage polarization, an event associated with the increase in NOX and the release of high amounts of pro-inflammatory cytokines. GSH: reduced glutathione; GSSG oxidized glutathione; NOX: NADPH oxidase; TRPM: transient receptor potential ion channels for melastatin; TXNIP: Thioredoxin Interacting Protein; ↑: increase; ↓: decrease.

Within the complexity and cellular heterogeneity of AT, it should also be recalled that low Mg promotes a pro-oxidant and pro-inflammatory phenotype in endothelial cells [73,74] (Figure 4). These data are further supported by the powerful effect of different Mg concentrations on endothelial transcriptome [75]. Specifically, low Mg markedly perturbs inflammatory pathways, events that adversely impact adipocyte metabolism, insulin sensitivity, and plasticity. Moreover, Mg reduces endothelial apoptosis in rats with preeclampsia by upregulating miR-218-5p, which targets the HMGB1 pathway, known to be pro-inflammatory [76] (Figure 4). Macrophages are also sensitive to Mg deficiency, which upregulates the M1 subtype markers and induces the secretion of inflammatory cytokines [77] (Figure 4). Briefly, Mg deficiency drives all cells to promote and maintain inflammation. The good news is that most of these detrimental effects are reversible when Mg homeostasis is restored.

While Mg is known to control transcription and translation [21], an aspect that is often neglected is its role in regulating chromatin dynamics [78]. Interestingly, Mg serves as a cofactor for methionine adenosyl transferase 1A (MAT1A), an enzyme responsible for producing S-adenosylmethionine (SAM). SAM is the primary methyl donor within cells and is crucial for numerous methylation reactions, including those involving DNA and proteins. Mg also influences the activity of enzymes that catalyze the methylation and demethylation of DNA, such as DNA methyltransferases (DNMTs) [79] and ten-eleven translocation (TET) enzymes [80]. Therefore, Mg emerges as a mineral linked to epigenetics, as demonstrated in pregnant rats, where Mg deficiency induces metabolic complications in their offspring by altering cytosine methylation in the promoter region of the hepatic hydroxysteroid dehydrogenase-2 gene [81], which contributes to fatty acid metabolism [82]. Recently, a proper intake of Mg has been proposed as a beneficial measure to prevent epigenetic changes that lead to impaired cardiovascular function [83]. At this point, a question arises: since epigenetic signatures are potentially reversible, are the reversible effects observed in vitro upon restoration of Mg homeostasis due to epigenetic regulation? More research is needed to find a proper answer. Understanding DNA methylation patterns in response to Mg is crucial for enhancing knowledge of potential prevention strategies by modifying nutritional status in at-risk populations.

## 6. Mg, Oxidative Stress, and Inflammation: Clinical Evidence

A search for articles on Mg and OS in AT or obesity resulted in a limited number of clinical studies investigating the role of Mg in preventing the generation of OS and inflammation in obese individuals. Some studies evaluated the links between Mg and anthropometric indices. Toprak et al. show that supplementing obese hypomagnesemic individuals (BMI > 30) with Mg oxide (365 mg die) for 3 months normalizes magnesemia, ameliorates metabolic profiles, and reduces waist circumference [84]. Indeed, a recent systematic review and meta-analysis of clinical trials concluded that Mg supplementation is associated with lower waist circumference only in obese subjects [85]. These results are in accordance with the data from the Mexican National Health and Nutrition Survey (ENSANUT), showing that increased dietary Mg intake is linked to lower body mass index and waist circumference [86]. On the contrary, higher Mg intake was not associated with differences in anthropometric indices in Iranian adults [87]. Of interest, increased fat mass is linked to lower Mg levels also in childhood obesity, suggesting that adipose tissue plays a crucial role in maintaining Mg balance [88]. Similar results were recently obtained in 189 Mexican schoolchildren [89].

At the same time, weight reduction may impact Mg levels. Mikalseni et al. [90] examined variations in serum Mg levels among obese individuals with and without diabetes following weight loss from dietary modifications and bariatric surgery. A moderate weight loss resulting from lifestyle changes caused a 5% increase in serum Mg levels in both diabetic and non-diabetic individuals. Following bariatric surgery, Mg levels remained stable in non-diabetic patients but continued to rise in diabetic patients. Six months after bariatric surgery, these two groups had no significant difference in serum Mg concentration. In obese individuals, diabetes is likely the primary cause of low Mg levels. Research by Lecube et al. [91] revealed that 48% of diabetic individuals and 15% of non-diabetic individuals were hypomagnesemic. There were notable negative correlations between Mg levels and fasting blood sugar, HbA1c, HOMA-IR, and BMI. Following bariatric surgery, serum Mg levels increased only in patients whose diabetes had resolved. However, there was no change in Mg levels among those who did not achieve glycemic control, with no discernible variations in weight loss outcomes between the two groups. Reduced Mg intake and increased urinary Mg loss are significant contributors to Mg deficiency in individuals with type 2 diabetes, while Mg absorption and retention seem to remain stable [92]. Both hyperglycemia and hyperinsulinemia can elevate urinary Mg excretion, whereas adequate metabolic control is linked to decreased Mg loss through urine. Mg also plays a crucial role in the regulation of insulin signaling. The clinical manifestation of a chronic Mg deficiency includes post-receptor insulin resistance, leading to decreased glucose utilization in cells. This exacerbates the existing insulin sensitivity reduction in type 2 diabetes and worsens hyperglycemia, potentially resulting in an elevated urinary excretion of Mg in a self-reinforcing cycle. Another potential connection between Mg deficiency and decreased insulin sensitivity lies in the existence of OS and LGCI, commonly prevalent in conditions correlated with Mg deficiency, including diabetes, hypertension, metabolic syndrome, and aging [92].

Pointing our attention to the links between impaired Mg homeostasis and inflammation, an analysis of the NHANES data from 1999–2002 on more than 10,000 adults found a 40% increase in elevated C-reactive protein (CRP), a hallmark of chronic inflammatory diseases, in individuals consuming less than the RDA for Mg [93]. Subjects consuming less than 50% of the RDA for Mg but supplementing with more than 50 mg per day were 22% less likely to have elevated serum CRP levels compared to those not taking a supplement [94].

Mg did not seem to exert any effect on systemic inflammatory markers, such as CRP, MCP-1, IL-6, and adiponectin [95] in 95 overweight and obese subjects supplemented with Mg glycinate 360 mg/die + vitamin D 1000 UI/die or only vitamin D 1000 UI/die vs. placebo for 12 weeks. Analogously, no effects on the levels of hs-CRP, IL-6, TNFα, sICAM-1, sVCAM-1, and E-selectin were observed in a cross-over study on 14 subjects supplemented with Mg citrate (500 mg elemental Mg/d) for 4 weeks [96]. On the contrary, a meta-analysis shows that Mg supplementation significantly reduces serum CRP levels [97]. Notably, the authors suggest designing RCTs with a larger sample size and a more extended follow-up period to give unequivocal answers [97]. More recently, a systematic review and meta-analysis has summarized the state of the art of 17 randomized control trials investigating the effects of Mg supplementation versus placebo on serum parameters of inflammation. The authors concluded that Mg supplementation significantly reduced plasma C reactive protein, fibrinogen, tartrate-resistant acid phosphatase type 5, tumor necrosis factor-ligand superfamily member 13B, ST2 protein, and IL-1 [98].

The relation between Mg and OS has been overlooked. In a study involving 23 individuals, Mg oxide supplementation (500 mg/die) for 28 days significantly decreased DNA oxidative damage of blood lymphocytes [99]. In a study on patients with hypertension, potassium and Mg citrate supplement (20 meq Mg = 243 mg per day) for 4 weeks brought a significant decrease in urinary 8-isoprostane, a stable end-product of lipid peroxidation, compared to placebo (13.5 ± 5.7 vs. 21.1 ± 10.5 ng/mg Cr) [100]. On the contrary, a RCT performed in women affected by polycystic ovary syndrome showed that 250 mg of Mg for 8 weeks did not produce an appreciable decrease or increase in total antioxidant capacity (TAC), despite a reduction achieved in the waist circumference of the subjects [101]. In a study involving children with atopic asthma, those aged 7 or younger received 200 mg of Mg citrate, while those older than 7 received 290 mg. The study found that after 12 weeks of treatment, there was a significant increase in reduced glutathione (GSH) concentration. However, there were no changes in the ratio of reduced to oxidized GSH, and no effects were observed on oxidized hemoglobin in plasma and whole blood [102]. The study conducted by de Oliveira and colleagues [103] examined the impact of Mg in mitigating OS among individuals with obesity using the levels of thiobarbituric acid-reactive substances (TBARS) in plasma and erythrocytes as markers of OS. The findings suggest that obese patients exhibit decreased Mg consumption in their diets, leading to hypomagnesuria as a compensatory response. While the plasma concentration of TBARS was notably higher in obese patients in comparison to the control group, there was no correlation between Mg levels and OS markers. In a study by Morais and colleagues [23], it was found that obese individuals tend to consume a low Mg diet, but this does not seem to affect their plasma and erythrocyte Mg levels. Additionally, the average plasma TBARS concentration was higher in obese women compared to the control group. However, it was observed that there is a negative correlation between Mg levels in erythrocytes and plasma TBARS, indicating the impact of Mg status on oxidative stress indicators in overweight women.

The studies collectively suggest Mg’s antioxidant and anti-inflammatory effects in various situations. However, they are limited in number and have been conducted on a small number of subjects. Additionally, baseline nutritional status is often not considered in supplementation studies. Supplementation of essential nutrients like Mg has a positive effect, especially in individuals with deficiencies (Table 2). In individuals with good nutritional status, it probably has no effect. Therefore, further well-designed studies are needed to elucidate the role of Mg in modulating OS in the whole body and obese AT.

**Table 2:** Clinical evidence of the effects of Mg supplementation on oxidative and inflammatory markers and insulin function.

Study	Year	Type of Trial	Mg mg Per Day	Mg Formulation	Timing of Administration (Weeks)	N° Subjects	Subjects’ Description	Effects
Cheung et al. [87]	2022	RCT DB, parallel	360	Mg glycinate	12	95	Healthy ow and ob 25 < BMI < 40	↑ in Vit D absorption and ↓ systolic BP, no effects on IL-6; MCP-1, adiponectin, and CRP
Toprak et al. [76]	2017	RCT DB, parallel	365	Mg oxide	12	128	Hypomagnesemic, pre-diabetic, ob with mild-to-moderate CKD	↓ of IR; HOMA-IR; HbA1c; insulin; WC and UA with an ↑ albumin and serum Mg level
Chacko et al. [88]	2011	RCT DB, cross-over	500	Mg citrate	4	14	Healthy ow BMI > 25	↓ fasting C-peptide and insulin; no effects on inflammatory markers
Petrovic’ et al. [91]	2016	CT, parallel	500	Mg oxide	4	23	Young male rugby student vs. sedentary student	↓ DNA oxidative damage in lymphocyte
Vongpatanasin et al. [92]	2016	RCT DB, cross-over	243	Potassium Mg citrate	4	30	Pre- or hypertensive subjects	↓ of urinary 8-isoprostane
Mousavi et al. [93]	2021	RCT DB, parallel	250	Mg oxide	8	84	PCOS women BMI < 35	No effect on TAC, ↓ CRP
Bede et al. [94]	2008	RCT DB, parallel	200–290	Mg citrate	12	40	Children with atopic asthma	↑ GSH, no effect on GSH/GSSG

BP = Blood Pressure; BMI = Body mass index; CKD = Chronic kidney disease; CRP = C-Reactive Protein; DB = Double Blind; HbA1c = Hemoglobin A1C; HOMA-IR = homeostatic model assessment for insulin resistance; GSH = reduced glutathione; GSSG = oxidized glutathione; IL-6 = Interleukin 6; IR = Insulin Resistance; MCP-1 = Monocyte Chemoattractant Protein-1; Mg = magnesium; ob = obese; ow = over-weight; PCOS = polycystic ovarian syndrome; RCT = Randomized Controlled Trial; TAC = total antioxidant capacity; UA = Uric Acid; WC = Waist Circumference; ↑ = increase; ↓ = decrease.

## 7. Conclusions and Future Directions

**Figure 5:**
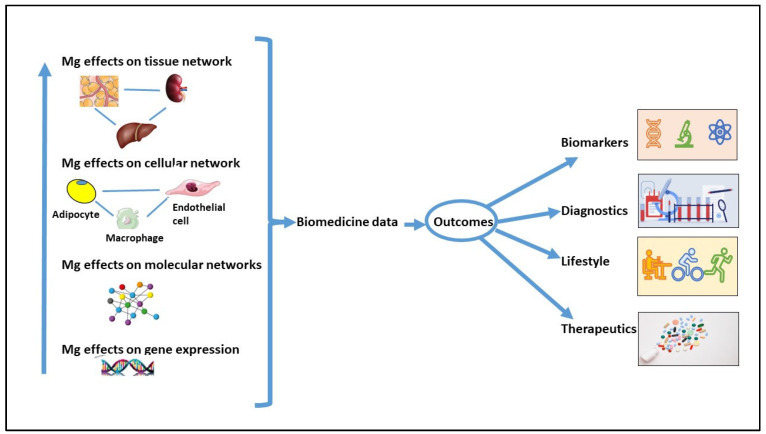
Biomedical research on Mg’s potential impact on health and disease prevention. Data from studies on the interaction between different cell types and between the various organs might result in the identification of biomarkers and strategies to prevent AT dysfunction and, in general, diseases.

In this narrative literature review, we highlight the role of Mg in regulating AT metabolism and its potential impact on preventing OS and LGCI in AT and obesity. This preventive action of Mg is primarily attributed to its role in maintaining mitochondrial function, supporting antioxidant defenses, and acting as a calcium antagonist. Nevertheless, despite extensive research in major databases, we found limited studies that directly or indirectly investigate these topics at preclinical and clinical levels. Studies should be conducted on the contribution of Mg in regulating adipogenesis and in modulating the function of adipocytes challenged with inflammatory cytokines and/or unbalanced levels of adipokines. New insights could be gained from omics studies conducted both in vivo and in vitro. This knowledge can contribute to the development of innovative treatment strategies to maintain health and prevent diseases (Figure 5). We hope that this review will inspire future research to delve deeper into the role of Mg in regulating metabolism and ROS production in AT.

## Abbreviations

adipose tissue (AT); brown adipose tissue (BAT); body mass index (BMI); calcium (Ca); cat-alase (CAT); C-reactive protein (CRP); endothelial cells (ECs); endothelial nitric oxide synthase (eNOS); European Food Safety Authority (EFSA); fatty acid synthase (FAS); fatty acyl transport protein 1 (FATP 1); glutathione (GSH); glutathione peroxidase (GPx); glutathione reductase (GR); heme oxygenase-1 (HO-1); insulin resistance (IR); interleukin (IL); low-grade chronic inflammation (LGCI); magnesium (Mg); mitochondrial RNA splicing 2 (MRS2); monocyte chemoattractant pro-tein-1 (MCP-1); NADPH oxidase (NOX); NAD(P)H: quinone oxidoreductase 1 (NQO1); nitric oxide synthase (NOS); nuclear factor-κB (NF-κB); oxidative stress (OS); paraoxonases (PON); perivascular adipose tissue (PVAT); peroxiredoxin (Prx); PPARγ coactivator 1-alpha (PGC1-α); PR domain containing 16 (PRDM 16); protein kinase C (PKC); reactive oxygen species (ROS); recommended dietary allowance (RDA); superoxide dismutase (SOD); thiobarbituric acid-reactive substances (TBARS); thioredoxin reductase (TrxR); Total Antioxidant Capacity (TAC); transient receptor po-tential ion channels for melastatin (TRPM); triglycerides (TG); tumor necrosis factor-α (TNF-α); white adipose tissue (WAT).
